# Does a coexisting congener of a mixed mating species affect the genetic structure and selfing rate via reproductive interference?

**DOI:** 10.1007/s00442-024-05607-x

**Published:** 2024-08-22

**Authors:** Koki R. Katsuhara, Atushi Ushimaru, Yuko Miyazaki

**Affiliations:** 1 Faculty of Environmental, Life, Natural Science and Technology, Okayama University, Tsushima-Naka 1-1-1, Kita-ku, Okayama, 700-8530 Japan; 2 Graduate School of Human Development and Environment, Kobe University, 3-11 Tsurukabuto, Kobe, 657-8501 Japan

**Keywords:** *Commelina*, Genetic diversity, Inbreeding coefficient, Mixed mating, Population genetics

## Abstract

Reproductive interference is defined as an interspecific interaction that reduces fitness via mating processes. Although its ecological and evolutionary consequences have attracted much attention, how reproductive interference affects the population genetic structures of interacting species is still unclear. In flowering plants, recent studies found that self-pollination can mitigate the negative effects of reproductive interference. Selfing-biased seed production is expected to increase population-level inbreeding and the selfing rate, and limits gene flow via pollinator outcrossing among populations. We examined the population genetics of the mixed-mating annual herb *Commelina communis* f. *ciliata*, focusing on reproductive interference by the sympatric competing congener *C*. *communis* using microsatellite markers. First, we found that almost all *C*.* c*. f. *ciliata* populations had relatively high inbreeding coefficients. Then, comparing sympatric and allopatric populations, we found evidence that reproductive interference from a competing congener increased the inbreeding coefficient and selfing rate. Allopatric populations exhibit varied selfing rates while almost all sympatric populations exhibit extremely high selfing rates, suggesting that population selfing rates were also influenced by unexamined factors, such as pollinator limitation. Besides, our findings revealed that reproductive interference from a competing congener did not limit gene flow among populations. We present the first report on how reproductive interference affects the genetic aspects of populations. Our results suggested that the high selfing rate of *C*.* c*. f. *ciliata* promotes its sympatric distribution with *C*. *communis*, even in the presence of reproductive interference, although it is not clear whether reproductive interference directly causes the high selfing rate.

**Supplementary Information:**

The online version contains supplementary material available at 10.1007/s00442-024-05607-x.

## Introduction

Reproductive interference, which is defined as any kind of interspecific interaction that reduces the fitness of both or either species via the mating process, is likely to occur between closely related species because these species often have similar reproductive biology (Gröning and Hochkirch 2008; Burdfield-Steel & Shuker 2011). Many recent studies have reported that reproductive interference plays an important role in ecological and evolutionary processes, for example by promoting competitive exclusion and character displacement (Gröning and Hochkirch 2008; Burdfield-Steel and Shuker 2011; Cothran 2015).

When two or more flowering plant species share a pollination niche, interspecific pollen transfer is thought to function as reproductive interference mainly via two processes: heterospecific pollen deposition on the stigma and conspecific pollen loss to heterospecific flowers (Harder et al. 1993; Morales and Traveset 2008; Mitchell et al. 2009). Heterospecific pollen transfer from closely related species substantially reduces seed production via stigma clogging (obstruction of a stigmatic surface), stylar clogging (physically crowding within the stylar tissue), ovule usurpation (ovule wasted by reaching prior to conspecific pollen tubes), and/or so on (reviewed in Morales and Traveset 2008; Moreira-Hernández and Muchhala 2019). Its negative effects increase with the relative abundance of the competitor (Levin and Anderson 1970; Katsuhara and Ushimaru 2019). Therefore, because the frequency of inferior species is supposed to be lower in the next generation due to reproductive interference in the current generation, its negative effect becomes greater in the next generation compared to the current generation. Thereby, such a positive feedback process strongly promotes competitive exclusion of the focal species (Levin and Anderson 1970; Kishi et al. 2009; Katsuhara et al. 2021). In Japanese dandelion species, reproductive interference is thought to be the main force driving displacement of native species by invasive species (Matsumoto et al. 2009; Nishida et al. 2014). Recent analyses of the distribution of the *Crepis* complex suggested that reproductive interference explains the spatially isolated distributions of sexual reproductive species (Whitton et al. 2017).

Some recent studies suggest that prior selfing can mitigate the negative effects of reproductive interference by close relatives in a given species, enabling them to coexist sympatrically (the pre-emptive hypothesis; Fishman and Wyatt 1999; Goodwillie and Ness 2013; Randle et al. 2018; Katsuhara and Ushimaru 2019; Katsuhara et al. 2021). Katsuhara and Ushimaru (2019) suggested that prior autonomous self-pollination might reduce the negative effect of reproductive interference, even with frequent pollinator visits in mixed-mating *Commelina* species. Theoretically, Katsuhara et al. (2021) demonstrated that two flowering species under the presence of reproductive interference can coexist via the co-evolution of higher rates of self-pollination in both species. Although these studies predicted an increased selfing rate under reproductive interference by congeners, especially in competitively inferior species, the extent to which selfing rates increase with coexisting congeners is largely unknown.

While much attention has been paid to the ecological and evolutionary consequences of pollinator-mediated reproductive interference, knowledge of the genetic consequences is still limited. Population genetics in species under reproductive interference from other species is an interesting topic in ecology and evolutionary biology, because increased selfing should influence their genetic structures, which in turn would determine the evolutionary and demographic dynamics of populations of such species (Hendry 2013; Katsuhara et al. 2021). Here, we predict the following changes in the genetic structure of a given population under reproductive interference from a coexisting congener population. First, the presence or increased abundance of a competing congener could increase the population selfing rate via increased selfing-biased seed production. While the pre-emptive selfing hypothesis predicts that higher selfing rate is promoted via evolutionary changes, selfing can also increase via immediate proximate effects of decreased outcrossing caused by reproductive interference (Katsuhara and Ushimaru 2019; Katsuhara et al. 2021). Second, reproductive interference from the competing congener is expected to decrease the size of the focal population due to decreasing seed sets (Katsuhara and Ushimaru 2019). Other than selfing, biparental inbreeding may also increase as population size declines (Ellstrand and Elam 1993; Angeloni et al. 2011). From the perspective of eco-evolutionary feedback, an increase in selfing or inbreeding could reduce the population growth rate by increasing inbreeding depression or lowering the potential for adaptive evolution due to decreased genetic diversity (Keller and Waller 2002; Charlesworth 2003; Angeloni et al. 2011). Finally, an increased selfing rate might limit gene flow among populations (Hamrick & Godt 1996; Ingvarsson 2002; Keller and Waller 2002; Dickinson et al. 2003).

Here, we examine the effects of reproductive interference on the population genetics of the mixed-mating annual herb *Commelina communis* f. *ciliata* (Ccfc) using microsatellite markers. Previously, we revealed that seed production by Ccfc decreased with increasing relative abundance of the sympatric congener *C*. *communis* (Cc), although prior autonomous selfing may ensure seed production (Katsuhara and Ushimaru 2019). We examined whether the seed production of individual Ccfc flowers was more selfing-biased when the relative abundance of a competing congener increased around them, and whether the genetic structure of Ccfc populations with a sympatric congener (sympatric populations) differed from those without (allopatric populations). We predicted higher inbreeding coefficients and population selfing rates, smaller effective population sizes, and higher inter-population genetic differentiation in sympatric than allopatric populations.

## Materials and methods

### Study system

*Commelina* L. is the largest genus (comprising about 170 species) in the family Commelinaceae (Faden 1998). *Commelina communis* f. *ciliata* (Ccfc) is distinguished from Cc by the presence of bract hair (Katsuhara and Ushimaru 2019; Katsuhara et al. 2019). Ccfc is usually 2*n* = 44 or 46 and Cc is usually 2*n* = 86, 88 or 90 (Fujishima 2003, 2010, 2017). The two taxa often grow sympatrically, but do not hybridise (Fujishima 2010; Katsuhara and Ushimaru 2019). Like Cc, Ccfc is andromonoecious of which perfect flowers exhibit a relatively high pollen/ovule ratio of 1300–1700 compared to 1000–1700 for Cc (Katsuhara and Ushimaru 2019). Our previous study has revealed Ccfc and Cc have largely overlapping habitats, flowering phenology, and pollinator composition (Katsuhara and Ushimaru 2019). Flowers of both species were visited by various pollinator species, such as *Bombus diversus diversus*, *Apis mellifera*, *Episyrphus balteatus*, and other hoverflies (Ushimaru et al. 2014; Katsuhara and Ushimaru 2019). Our observation has demonstrated that inter-species flower movements by pollinators occur according to the relative frequency of flowers probably because pollinators did not discriminate between Ccfc and Cc flowers (Katsuhara and Ushimaru 2019). It results in both species suffering mutual frequency-dependent reproductive interference in sympatric populations, i.e. the seed production of each species decreases with an increase in the relative flower abundance of the competing species (Katsuhara and Ushimaru 2019).

We examined 12 allopatric and 10 sympatric populations, comprising only Ccfc individuals and Ccfc individuals sympatrically distributed with Cc, respectively (Table S1). In 2017, a fresh leaf was sampled from Ccfc individuals in three allopatric (A01–A03) and sympatric (S01–S03) populations. Leaves were sampled from seven allopatric and nine sympatric populations in 2020 (see Table S1) and genotyped with 10 microsatellite markers (YP28, YP31, and YP33 from Li et al. 2015; Ccfc01, Ccfc05, Ccfc09, Ccfc25, Ccfc28, Ccfc31, and Ccfc32 from Katsuhara et al. 2019) for population genetic analyses.

The genotypes of sampled leaves were characterised as follows. Genomic DNA was extracted from each sample using the CTAB method (Murray and Thompson 1980). PCR amplification was performed in 12 μL volumes, including approximately 5 ng template DNA, 6 μL 2× Multiplex PCR Master Mix (QIAGEN) and primers (0.1 μM forward, 0.2 μM reverse, and 0.1 μM M13 fluorescently labelled primers; Boutin-Ganache et al. 2001). The thermal treatment was as follows: initial 15-min denaturation at 95°C; 35 cycles of 94°C for 30 s, 57°C for 1.5 min and 72°C for 1 min; and a final 30-min extension at 60°C. The PCR products were measured using the ABI3730XL DNA analyser and Genotyper software Peak scanner (Applied Biosystems).

### Relationship between the selfing rate and relative abundance of the competing congeners

We conducted three field surveys each for populations S01–S03, in the period September 4–25, 2017. Each survey used 4–6 2 × 2 m^2^ plots. In each plot, we counted Ccfc and Cc flowers to determine their relative abundances, and arbitrarily selected and marked 1–5 flowers from each of 1–5 individuals to sample leaves and seeds. Pollinator visits on Ccfc flowers were observed for 45 min per plot to estimate the pollinator abundance, defined as the visit frequency per flower per hour for each day and population.

About 1 month after the flowering season, we sampled a seed from the marked flower, where a single flower produces four seeds at most: some marked flowers were lost. We genotyped embryos, which were removed from the sampled seeds carefully using a scalpel. When an embryo had alleles absent from the parent leaf, the seed was defined as an outcrossing seed. Otherwise, we defined the seeds as selfing seeds. For the subsequent analysis, we estimated the outcrossing rate as the number of outcrossing seeds divided by the total number of seeds in the focal plot. This estimate was very conservative, and the number of selfing seeds might have been over-estimated, especially in populations with lower effective population sizes, because the outcrossed pollen could have the same alleles as the seed parent.

We conducted generalised liner mixed model (GLMM) analysis (binomial error and logit link). In the model, the outcrossing rate in the focal plot was included as the response variable, and the relative flower abundance of the competing species (i.e. Cc flower number/total Commelina flower number), pollinator abundance, and their interaction in the focal plot were the explanatory variables. The observation date and population identities were included as independent random terms. The GLMM analysis was performed using R, with the glmmADMB package (ver. 4.0.2; R Core Team 2020) (Fournier et al. 2012).

### Comparison between allopatric and sympatric populations

To examine the effect of the competing species on the genetic structure of Ccfc populations, we calculated genetic characteristics and compared them between sympatric and allopatric populations. All indicators were calculated using the genotype data of the sampled leaves with 10 microsatellite markers. A leaf was sampled from different individuals in each population, and the sample number per population varied from 11 to 32 depending on the population size (Table S1).

We calculated the following indices using FSTAT (Goudet 2003): mean number of alleles per locus (*A*), allelic richness (AR), number of rare alleles (RA), and inbreeding coefficient (*F*_IS_). Rare alleles were defined as those with relative frequencies < 1% in all study populations. The observed heterozygosity (*H*_O_) and Nei’s unbiased expected heterozygosity (*H*_E_) were calculated with GenAlEx (Peakall and Smouse 2006; 2012). A population’s selfing rate was estimated using three methods: classical estimation based on the inbreeding coefficient, where *S*_F_ = 2 *F*_IS_/(1 + *F*_IS_) (Wright 1969); S_RMES_, estimated based on the distribution of multilocus heterozygosity using the program RMES (David et al. 2007); and S_SIB_ estimated based on the sibship assignment method of the COLONY program (Jones and Wang 2010; Wang and Scribner 2014). Our hypothesis predicts reproductive interference enforces selfing-biased seed production which results in higher inbreeding coefficients and population selfing rates in sympatric populations. However, it should be noted that these indices cannot discriminate between autonomous and pollinator-mediated selfing (geitonogamy and facilitated selfing). The effective population size of each population was also estimated as N_e_, _LD_, N_e_, _Cn_, and N_e_, _SIB_ based on the linkage disequilibrium, molecular co-ancestry, and sibship assignment methods, respectively (Hill 1981; Nomura 2008; Wang 2009; Waples and Do 2010). N_e_, _LD_ and N_e_, _Cn_ were calculated using NeEstimator (Do et al. 2014) and N_e_, _SIB_ was calculated using COLONY (Jones and Wang 2010). The significance of the difference in these indices was tested by calculating the bootstrap *p*-value based on 10,000 times resampling (Fig. S1). As alternative hypotheses, sympatric populations were assumed to exhibit higher inbreeding coefficient (*F*_IS_) and population selfing rates (S_F_, S_RMES_, S_SIB_), and lower genetic diversities (A_R_ and H_E_) and effective population sizes (N_e, LD_, N_e, Cn_, N_e, SIB_). The statistical powers were also tested by bootstrap resampling because the number of sample populations using tests in some indices is low due to the limitation of estimating methods (see Fig. S2 for the details).

To examine genetic differentiation between populations, we calculated pairwise Jost’s estimate of differentiation (Jost’s D) values using the mmod R package (Jost 2008; Winter 2012). To determine whether sympatric population pairs had greater genetic differentiation than allopatric population pairs, we used a GLM that included pairwise Jost’s D as the response variable, pairwise types (allopatric *vs*. allopatric, allopatric *vs*. sympatric, and sympatric *vs*. sympatric) as the explanatory variables, and pairwise Euclidean distances (km) as covariates. The GLM was then constructed, and the significance of the estimated coefficients was determined by a permutation test performed using the lmPerm R package (Wheeler and Torchiano 2010). Then, to visualise genetic differentiation among the 22 populations, we performed Bayesian-based clustering using STRUCTURE with no prior information on population origins (Pritchard et al. 2000). Ten independent runs each for *K* = 1–25 were performed with a burn-in period of 100,000 steps followed by 100,000 Markov chain Monte Carlo iterations. We determined the optimal number of clusters (K) based on ∆*K* (Evanno et al. 2005) using STRUCTURE HARVESTER (Earl and von Holdt 2012).

## Results

### Relationship between the selfing rate and relative abundance of competing congeners

**Fig. 1:**
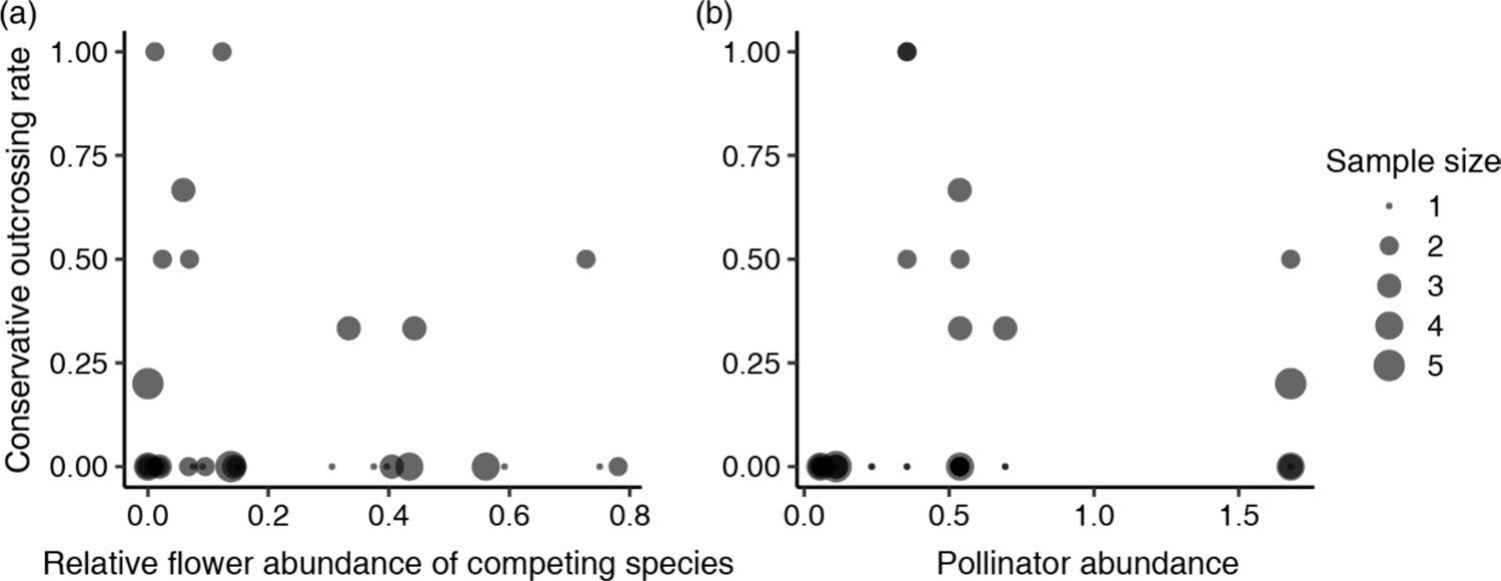
Relationships **a** between the relative flower abundance of competing species (*Commelina communis*) and conservative outcrossing rate of *C*.* c*. f. *ciliata*, and **b** between pollinator abundance and the conservative outcrossing rate. The circle size indicates the sample size (the number of flowers sampled in a focal plot). Generalised linear mixed model analysis showed no significant correlations between the conservative outcrossing rate and the relative flower abundance of competing species or pollinator abundance

In total, we genotyped 77 leaves and 77 seeds, and identified 12 seeds that were outcrossed (7, 5, and 0 in populations S01–S03, respectively). In the GLMM analysis including the conservative outcrossing rate as the response variable, we found no significant relationships between the rate and explanatory variables (relative flower abundance of competing species, *z* value = –1.40, *p* value = 0.16; pollinator abundance, *z* value = –0.65, *p* value = 0.52; interaction term, *z* value = 1.69, *p* value = 0.09; Fig. 1). A high outcrossing rate was seen with a lower relative flower abundance of the competing species and moderate pollinator abundance, although the result was not significant (Fig. 1).

### Comparison between allopatric and sympatric populations

**Fig. 2:**
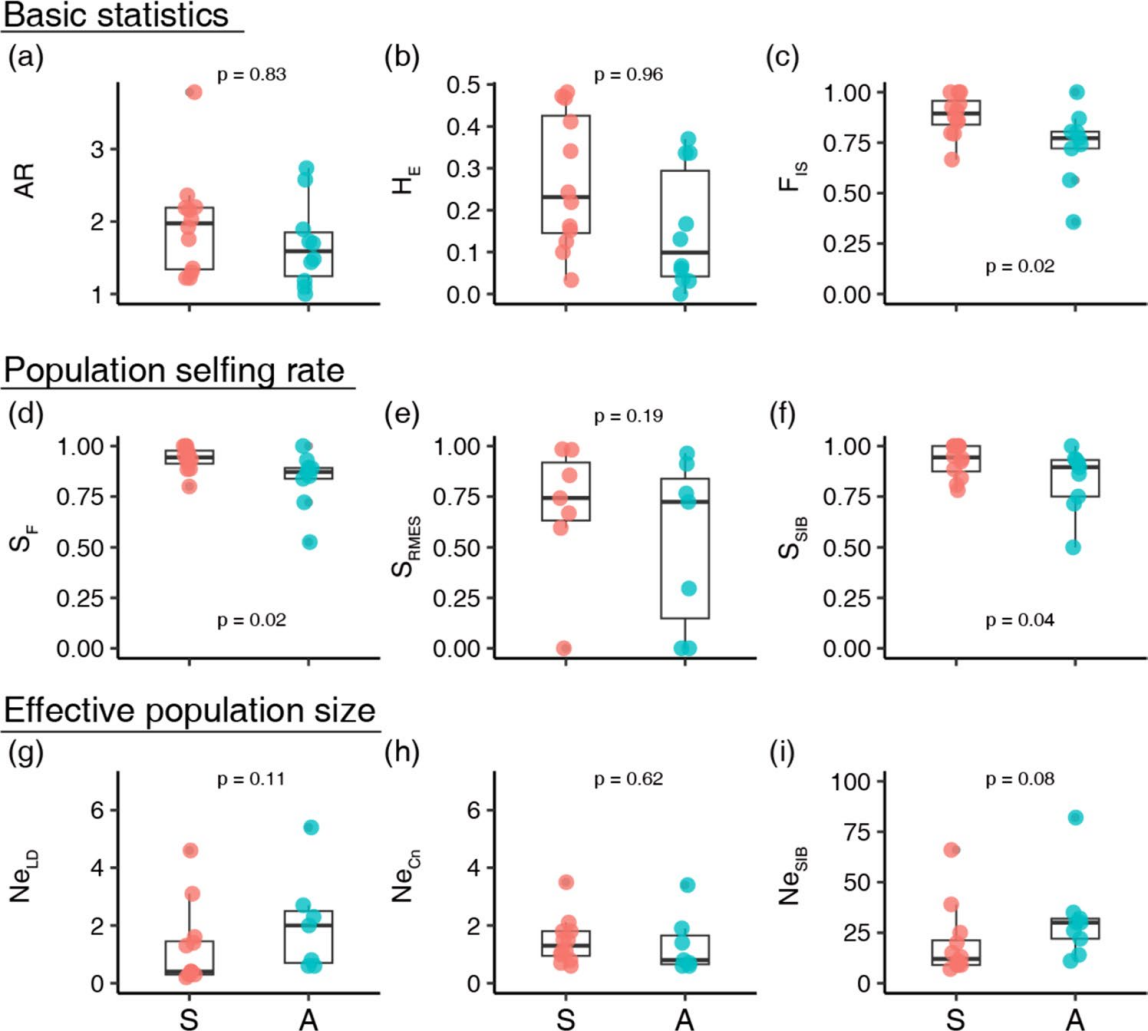
Boxplots of the genetic indices of sympatric (S) and allopatric (A) populations: **a** allelic richness (AR); **b** Nei’s unbiased expected heterozygosity (H_E_); **c** inbreeding coefficient (F_IS_); **d** population selfing rate based on the inbreeding coefficient (S_F_); **e** population selfing rate based on distributions of multilocus heterozygosity (S_RMES_); **f** population selfing rate based on sibship assignment methods (S_SIB_); and effective population size based on **g** linkage disequilibrium (N_e_, _LD_); **h** the molecular co-ancestry (N_e_, _Cn_); and **i** sibship assignment methods (N_e_, _SIB_). The bootstrap *p* values were shown in each panel; sympatric populations had significant higher F_IS_, S_F_, and S_SIB_ compared to allopatric populations

In total, we characterised 267 and 234 Ccfc individual genotypes in sympatric and allopatric populations, respectively (Table S1). Both population types had comparable genetic diversity in terms of AR and *H*_E_ (Figs. 2, S1). Numbers of rare alleles (RA) were also comparable in sympatric (mean and range are 2.0, and from 0 to 7) and allopatric (2.0, from 0 to 5) populations (Table S1). Sympatric populations tended to have significantly higher *F*_IS_ than allopatric populations, and both population types had relatively high mean *F*_IS_ values (0.89 and 0.74 in sympatric and allopatric populations, respectively; (Figs. 2, S1). Regarding the population selfing rate, mean *S*_F_ and *S*_SIB_ were significantly higher in sympatric than allopatric populations, while *S*_RMES_ did not differ significantly between allopatric and sympatric populations (Figs. 2, S1). Our statistical power analyses showed that no significance for S_RMES_ was likely due to the low sample size because *S*_RMES_ could not be estimated in some populations (Fig. S2). Note that *F*_IS_ and *S*_F_ are mathematically mutually related. Regarding the effective population size, all indices (*N*_e_, _LD_, *N*_e_, _Cn_ and *N*_e_, _SIB_) did not differ significantly between population types (Figs. 2, S1).

**Fig. 3:**
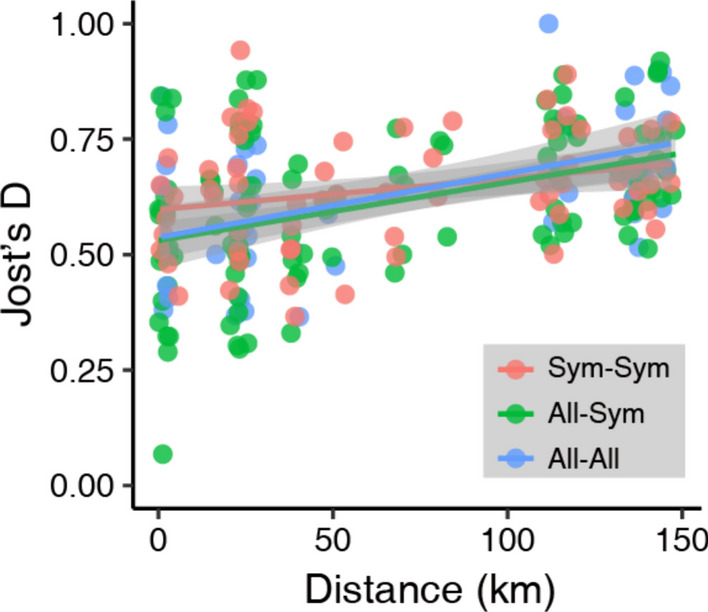
Relationships between the pairwise Euclidean distance and pairwise Jost’s estimate of differentiation (Jost’s D) values. The colours of the circles and lines indicate sympatric *vs*. sympatric (red), allopatric *vs*. sympatric (green), and allopatric *vs*. allopatric (blue). Generalised linear model analysis showed significant positive correlations between the pairwise distance and Jost’s D, while pairwise type had no significant effect on the pairwise Jost’s D. The regression lines and their confidence intervals were drawn based on the generalised linear models

**Fig. 4:**
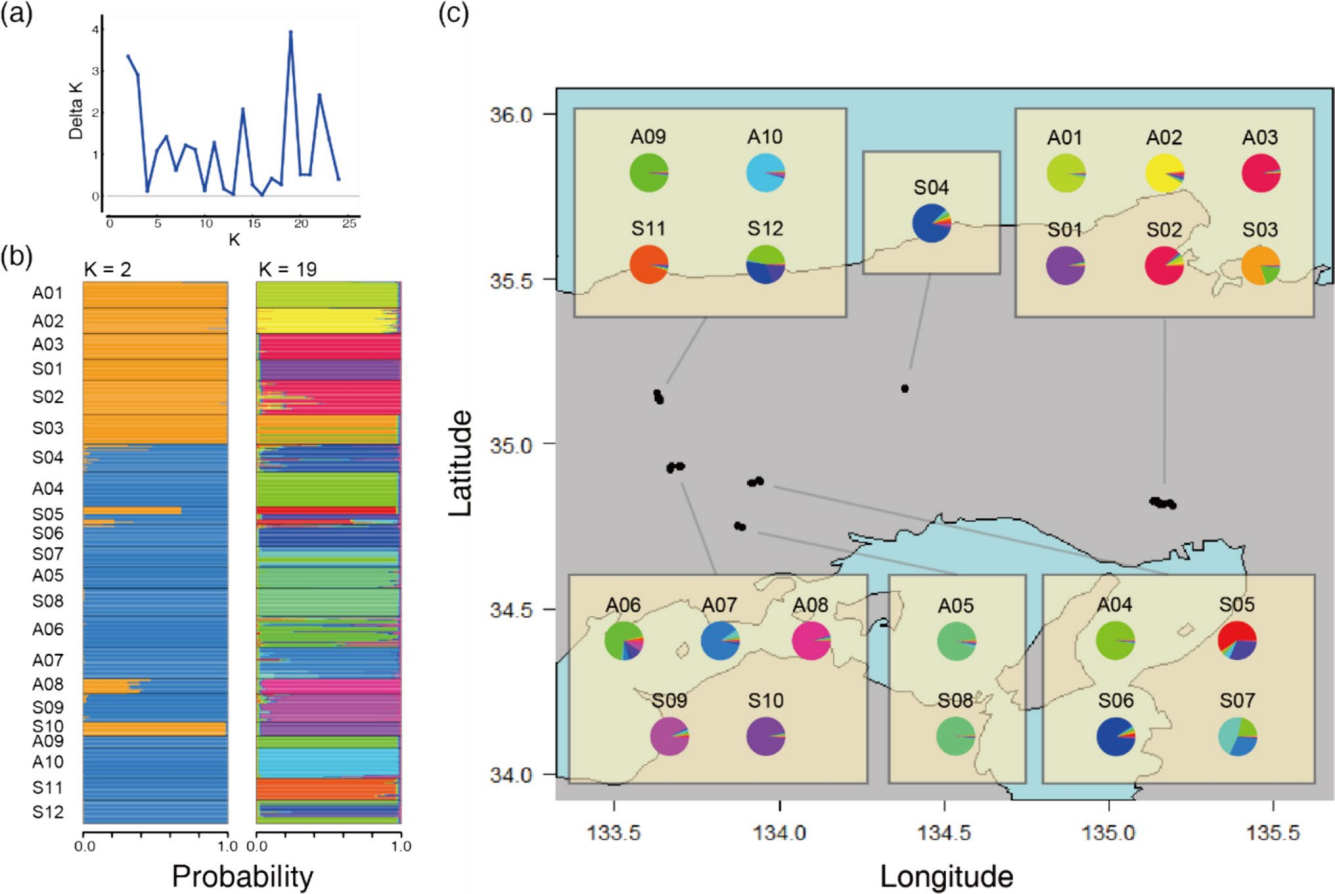
Results of the STRUCTURE analysis: **a** values of ΔK, based on the rate of change in ln P(X/K) between successive *K* values generated from STRUCTURE HARVESTER; **b** bar plots of STRUCTURE analyses where *K* = 2 and 19; **c** pie plots of the STRUCTURE analysis where *K* = 19 and locations of each population. The bar and pie plots indicate the probability of a sample being assigned to each cluster; clusters are represented by different colours

We found that all pairwise populations had relatively high Jost’s D values, and pairwise type had no significant effect on the mean values (0.64, 0.61, and 0.64 in the sympatric *vs*. sympatric, allopatric *vs*. sympatric, and allopatric *vs*. allopatric comparisons, respectively; Fig. 3). The pairwise Jost’s D value was positively correlated with the pairwise Euclidean distance (estimated coefficient and permutation *p*-values of 0.001 and < 0.001, respectively). Our STRUCTURE HARVESTER analysis indicated that ∆*K* was maximal when *K* = 19. The results obtained from STRUCTURE suggested that almost all populations belonged to a specific genetic cluster, while A03 and S02, A05 and S08, and S01 and S10 were suggested to belong to the same genetic clusters (Fig. 4).

## Discussion

We found that populations with sympatric congeneric species had higher population selfing rates and inbreeding coefficients than allopatric populations. For all three indices of the population selfing rate, sympatric populations had similarly high values, while those of allopatric populations varied from low to high, although the mean value did not differ significantly for S_RMES_. The population selfing rate might be influenced by factors other than reproductive interference from Cc, such as pollinator limitation; a higher population selfing rate might be favoured even in some allopatric populations with pollinator limitation, while higher selfing values are always favoured in populations in the presence of reproductive interference with or without pollinator limitation (Eckert et al. 2010; Katsuhara et al. 2021). In the sympatric populations, we found no significant correlation between the outcrossing rate of each flower and the relative flower abundance of competing species. A high outcrossing rate was observed only when flowers were surrounded by competing species with very low levels of flower abundance, although this result was not significant (Fig. 1). This result might be due to the Ccfc flowers in sympatric populations producing almost all of their seeds via selfing or inbreeding, independent of the relative flower abundance of competing species at the micro-spatial scale.

Our results suggested that coexisting with a competing congener did not decrease the effective population size of the focal species and genetic diversity, being am unexpected result based on the findings of our previous study showing that seed production of Ccfc flowers decreased with an increase in surrounding Cc flowers (Katsuhara and Ushimaru 2019). It seems that there are other factors that influence Ccfc population dynamics, such as disturbance and/or seedling competition. Some studies reported Cc exhibits seed dormancy, suggesting that seed banks could contribute to maintaining population genetic diversity (Takabayashi and Nakayama 1978; Yang et al. 2018). In Ccfc, seed banks likely can mitigate the effect of reproductive interference on effective population size and genetic diversity, which is a challenging subject and must be examined in future studies.

Both sympatric and allopatric Ccfc populations had high genetic differentiation from each other, even when these populations were close together, although genetic differentiation increased with the geographic distance between populations [mean Jost’s D 0.54 (range 0.07–0.84), even when the pairwise population distance < 10 km]. The results of the STRUCTURE analyses also showed that populations in the same region often belong to different genetic clusters. Because the seed dispersal mode of Ccfc is barochory (gravity), gene flow among populations is thought to depend mainly on pollinator-mediated pollen transfer. Although our results did not directly support reproductive interference from the competing congener limiting gene flow among populations, high selfing rate in our study populations might explain the high genetic differentiations among them.

In conclusion, the findings provided some support for our hypothesis that reproductive interference has genetic consequences, such as an increased population selfing rate and inbreeding. To our knowledge, this is the first report to address how reproductive interference affects population genetic structure in the field. In the future, three questions should be addressed. First, what floral traits are related to an increased selfing rate? Large and blue petals, heteranthery, and frequent pollinator visits to Ccfc flowers are inconsistent with the highly selfing-based reproduction revealed by our study. Comparing floral traits related to self-pollination, such as herkogamy (the distance between the anther and stigma; Webb and Lloyd 1986) and dichogamy (the time separation of pollen and stigma presentation; Lloyd and Webb 1986) between sympatric and allopatric populations might be important for understanding the evolutionary consequences of reproductive interference. Second, how does reproductive interference interact with other factors, such as pollinator limitation and inbreeding depression, to determine the genetic structure in species that enables selfing? In the presence of reproductive interference from a competing congener, not only pollinator limitation but also frequent pollinator visits might promote to increase selfing rate (Katsuhara et al. 2021). Finally, how can we determine whether a high population-level selfing rate is a requirement for or consequence of coexistence with competing congeners? This is a very challenging issue. In other words, instead of considering secondary contact in which the population with a high selfing rate under pollinator-limited conditions, we are interested in whether the rapid evolution of self-pollination driven by pollinator-mediated reproductive interference enables coexistence. Experimental studies focusing on eco-evolutionary dynamics are needed to answer this question; our field survey suggests a new basis for the relationship between population genetic structure and reproductive interference.

## Supplementary Information

Below is the link to the electronic supplementary material.

Table S1. Population localities and indices for the genetic structure of studied populations. Fig. S1. The detail of bootstrap tests to compare genetic indices between sympatric and allopatric populations. Fig. S2. The relationships between sample sizes and the statistical powers in bootstrap tests. Supplementary file1 (DOCX 661 KB)

Supplementary file2 (XLSX 15 KB)

## Data Availability

The datasets used during the current study are available in Zenodo (10.5281/zenodo.7002785).
