# Supplementary material for: Does a coexisting congener of a mixed mating species affect the genetic structure and selfing rate via reproductive interference?
Source: Oecologia. 2024 Aug 22;206(1-2):37–45. doi: 10.1007/s00442-024-05607-x (PMC11489367; doi:10.1007/s00442-024-05607-x)
Supplement: Supplementary file 1 — Table S1. Population localities and indices for the genetic structure of studied populations. Fig. S1. The detail of bootstrap tests to compare genetic indices between sympatric and allopatric populations. Fig. S2. The relationships between sample sizes and the statistical powers in bootstrap tests. Supplementary file1 (DOCX 661 KB) [file 442_2024_5607_MOESM1_ESM.docx]

**
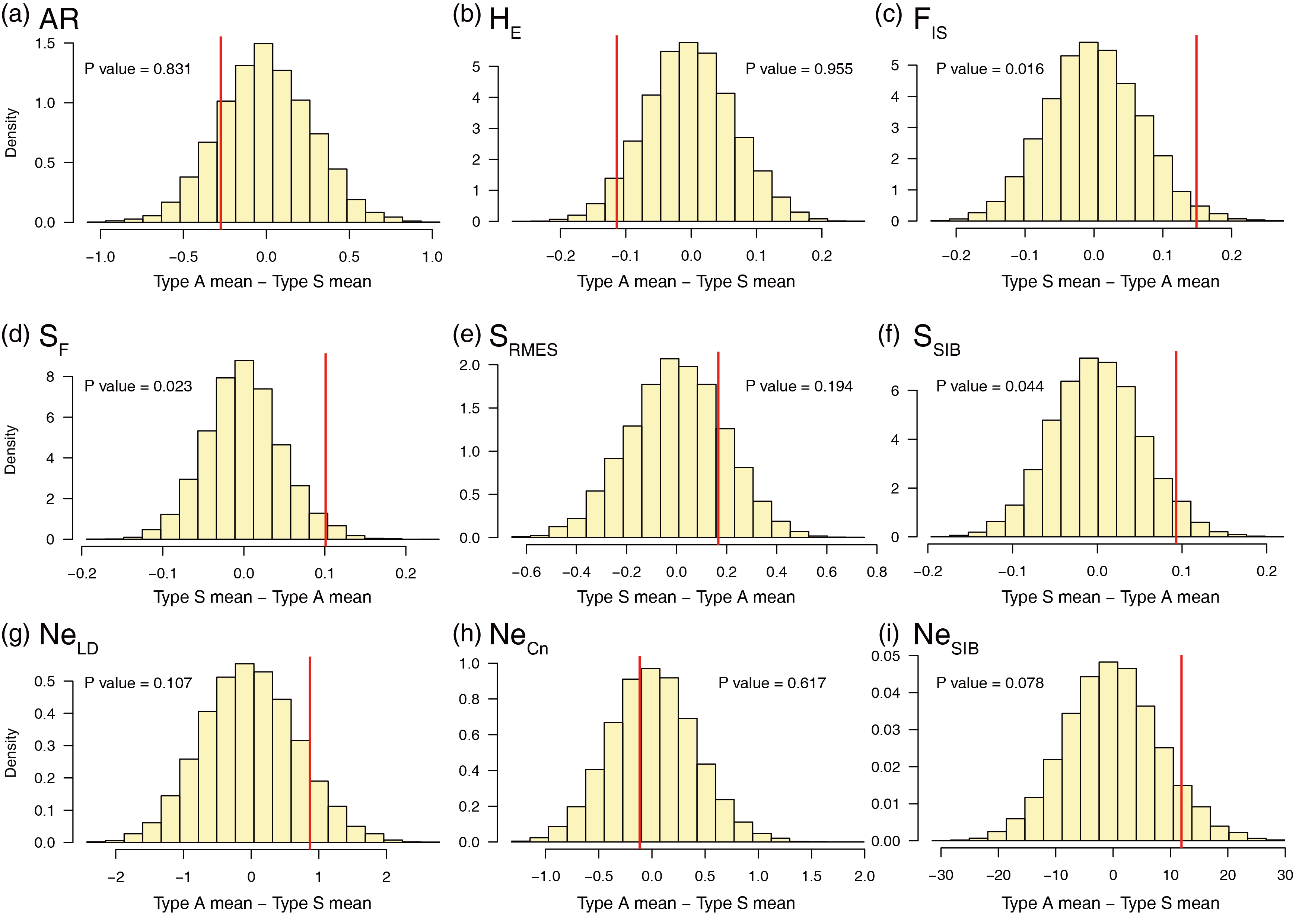
**

**Fig S1.** The null distributions of the expected difference in nine genetic indices between sympatric and allopatric populations based on 10,000 times bootstrap resampling: (a) allelic richness (AR); (b) Nei’s unbiased expected heterozygosity (H_E_); (c) inbreeding coefficient (F_IS_); (d) population selfing rate based on the inbreeding coefficient (S_F_); (e) population selfing rate based on distributions of multilocus heterozygosity (S_RMES_); (f) population selfing rate based on sibship assignment methods (S_SIB_); and effective population size based on (g) linkage disequilibrium (N_e_, _LD_); (h) the molecular co-ancestry (N_e_, _Cn_); and (i) sibship assignment methods (N_e_, _SIB_). Red lines indicated observed values.

**Methods:** To examine the significance of differences in genetic indices between sympatric and allopatric populations, we calculated their bootstrap *p*-value. We assumed that sympatric and allopatric populations had no difference in all genetic indices as the null hypothesis. For genetic diversities (AR and H_E_) and effective population sizes (N_e_, _LD_, N_e_, _Cn_, and N_e_, _SIB_), we expect that these metrics show higher values in allopatric than in sympatric populations as the alternative hypothesis. By contrast, inbreeding coefficient (F_IS_) and population selfing rate (S_F_, S_RMES_, and S_SIB_) are expected to be higher in sympatric than in allopatric populations as the alternative hypothesis. Null distributions of the difference in each index value between sympatric and allopatric populations were generated based on the null hypothesis, such that we randomly assigned the index values from all study populations to sympatric and allopatric populations while keeping the original sample size for each, and then calculated the mean difference in the value between pairs of sympatric and allopatric populations. The procedure was repeated 10,000 times to make null distribution. Then, bootstrap *p*-values were calculated as the proportion with values greater than the observed value within the null distribution. If the *p*-value < 0.05, it means the difference is significant in one side test with α = 0.05.

**Results:** We found that the inbreeding coefficient (F_IS_) and two indices for population selfing rate (S_F_ and S_SIB_) were significantly higher in sympatric than in allopatric populations while other indices exhibited no significant differences between population types. All bootstrap *p*-values were shown in Fig S1.

**
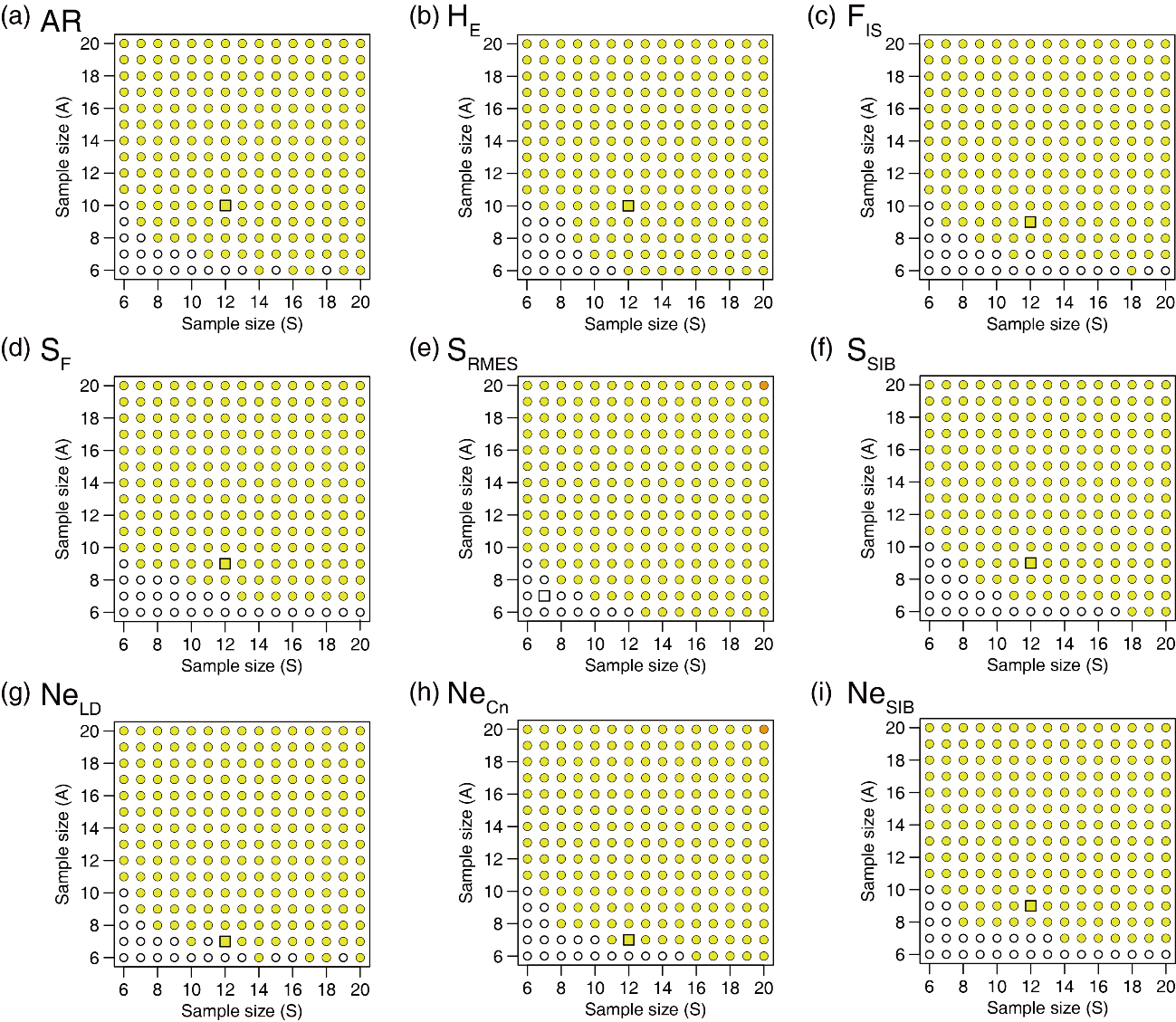
**

**Fig S2.** The relationships between sample sizes and the statistical powers in the bootstrap test, of which detail was described in the below method. The axes indicate the number of resampled populations of allopatric (A) and sympatric (S) in the bootstrap simulations. Filled and open circles indicate that the bootstrap test provided significant differences or not, respectively. Yellow and orange circles mean that the bootstrap tests detected statistically significance difference between the two population types, assuming a large effect size (Cohen’s d = 0.8) or moderate effect size (Cohen’s d = 0.5), respectively. The circles are replaced with squares when the sample size is the same as we observed actually (i.e. original sample size). Panels showed results of (a) allelic richness (AR); (b) Nei’s unbiased expected heterozygosity (H_E_); (c) inbreeding coefficient (F_IS_); (d) population selfing rate based on the inbreeding coefficient (S_F_); (e) population selfing rate based on distributions of multilocus heterozygosity (S_RMES_); (f) population selfing rate based on sibship assignment methods (S_SIB_); and effective population size based on (g) linkage disequilibrium (N_e_, _LD_); (h) the molecular co-ancestry (N_e_, _Cn_); and (i) sibship assignment methods (N_e_, _SIB_).

**Methods:** We conducted the power analysis for our bootstrap test to confirm whether our sampling sizes were enough to detect the statistical significance. First, we calculated an expected value of difference in a given index between sympatric and allopatric populations by assuming these two population types have different mean values. Here, we delivered the expected differences in each index based on the typical measurement of effect size, Cohen's d, as follows;

$Expected difference in the index= {Cohen}^{'}s d* Standard deviation$.

We calculated the standard deviation based on unbiased sample variance of a given index among all populations. Cohen's d was assumed 0.8 and 0.5, generally meaning the effect size is large and moderate, respectively. Next, we conducted the bootstrap test as we explained in Fig. S1, and investigated whether the test can detect the significance when we observed the expected difference in each index with the assumption that two population types have largely (Cohen's d = 0.8) and moderately (0.5) different mean values. Null distributions of difference between two population types, generated by 10,000 times resampling, were made with the assumption of various sample sizes from smaller (6 for each population type) to larger (20 for each population type) than the original sample sizes. Then, we calculated bootstrap *p*-values as the proportion of which null distribution exceed the expected difference. With this method, we tested whether our sample sizes were enough to detect significant difference in each index between the two population types.

**Results:** For all indices other than S_RMES_, we found our bootstrap tests with the original sample size were enough to detect the significance of difference when the effect size is large (Cohen's d = 0.8). When the effect size is moderate (Cohen's d = 0.5), the significance of difference cannot be detected in most cases and the bootstrap tests with a large sample size (N = 20) showed the detection of significance only for S_RMES_ and N_e_, _Cn_. As we provided in the main text and Fig. S1, our bootstrap tests revealed the observed differences in F_IS_, S_F,_ and S_SIB_ between sympatric and allopatric populations were significant while that in S_RMES_ was not significant. Our power analyses successfully showed that the differences in F_IS_, S_F_ and S_SIB_ were significantly large and that in S_RMES_ was non-significant due to the small sample size although it might actually be significant.
